# Recycling: Funds for Phones

**Published:** 2004-10

**Authors:** Richard Dahl

Americans will retire about 100 million cell phones this year when they switch to new models or new carriers, according to INFORM, a nonprofit environmental research group. Many go into the trash, ending up in landfills, and still more are tossed into closets and drawers, where they await the same ultimate destination. As University of Florida researchers concluded in the July 2004 report *RCRA Toxicity Characterization of Computer CPUs and Other Discarded Electronic Devices*, cell phones often release enough lead under test conditions to be classified as hazardous waste under federal law [see “Electronics, Lead, and Landfills,” *EHP* 112:A734 (2004)]. But while this growing mountain of old phones is drawing the attention of people who are worried about its potential impact on the environment, it has also been discovered by people who see it as a commodity that still retains market value.

In recent years, companies have emerged that buy old cell phones from individuals or groups that collect them on fundraising drives. These companies then sell the phones to foreign wireless carriers, who refurbish and resell them, or recyclers, who extract metals such as gold, silver, and copper. One company, RMS Communications Group, is currently taking in about 80,000 phones per month, according to its marketing and communications manager, Lynda Gorsuch. Like many such companies, RMS emphasizes charity tieins, offering people the opportunity to make a “virtual contribution” of the dollar value of the phone they sent in to a variety of charities listed on the RMS web-sites **http://www.cellforcash.com/** and **http://www.wirelessfundraiser.com/**. Another such firm is CollectiveGood (**http://www.collectivegood.com/**), which offers a similar smorgasbord of charities to which people can contribute the value of their old phones.

Meanwhile, youth organizations have found that cell phone collection drives offer unparalleled fundraising opportunities. On Earth Day 2004, a group of Boy Scouts in West Jordan, Utah, collected cell phones that they sold to RMS. RMS paid the Scouts from a set price list ranging from $3 to $100 per phone. The public response was so overwhelming that the Scouts are continuing the program and expect to make $6,000 from it this year, says David Bresnahan, an adult advisor who set up the project.

“We’re trying to promote environmental protection, which is a great lesson for the kids,” Bresnahan says, “and we’re putting the money into a fund that’s used to help kids who can’t afford to go to camp or can’t afford a backpack.”

A 2003 INFORM report, *Calling All Cell Phones*, analyzed various U.S. cell phone collection and recycling programs and concluded that, while they are providing a “critically important” service, they are not nearly enough. INFORM senior researcher Bette Fishbein says industry programs will absorb about 1% of this year’s discarded phones, and independent programs such as RMS and CollectiveGood will absorb a bit more. But the total amount of phones being taken in is still well under 5% of the 100 million that will be discarded, she says.

“It’s a step in the right direction,” Fishbein concludes. “But if you’re going to address the issue of toxics entering our environment through disposal facilities, you’ve got to take back a lot more.”

